# FibroidX: Vision Transformer-Powered Prognosis and Recurrence Prediction for Uterine Fibroids Using Ultrasound Images

**DOI:** 10.3390/cancers18040605

**Published:** 2026-02-12

**Authors:** Fatma M. Talaat, Yathreb Bayan Mohamed, Amira Abdulrahman, Mohamed Salem, Mohamed Shehata

**Affiliations:** 1 Faculty of Artificial Intelligence, Kafr Elsheikh University, Kafr Elsheikh 33516, Egypt; amira_644a@ai.kfs.edu.eg (A.A.); mohamed_726a@ai.kfs.edu.eg (M.S.); 2 School of Public Health and Information Science, University of Louisville, Louisville, KY 40208, USA; ybnajd01@louisville.edu; 3 Computer Science Program, James C. Bowling School of Business, Midway University, Midway, KY 40347, USA

**Keywords:** uterine fibroid, explainable artificial intelligence, pharmacological therapy, recurrence prediction

## Abstract

Background/Objectives: One of the common gynecological issues that can have a major effect on women’s reproductive health and quality of life is uterine fibroids (UFs). For personalized treatment planning and a reduction in long-term consequences, early fibroid prognosis and recurrence prediction are essential. In this context, prognosis refers to anticipated symptom progression and treatment response, while recurrence prediction estimates the likelihood of regrowth after interventions such as myomectomy, uterine artery embolization (UAE), or new fibroid formation during follow-up. Conventional techniques for predicting the prognosis and recurrence of UFs depend on imaging, clinical evaluations, and statistical models; nevertheless, they frequently have limited accuracy and are subjective. Methods: Therefore, we introduce FibroidX, which utilizes vision transformers and self-attention processes to improve forecast accuracy, automate feature extraction, and offer customized risk evaluations to overcome these obstacles. Prognosis encompasses overall disease progression, symptom severity, and response to therapy, whereas recurrence prediction focuses on post-treatment regrowth or new fibroid formation. Results: The dataset comprises 1990 ultrasound images split into training-test sets (80-20). With an accuracy of 98.4%, the suggested model outperformed baseline models like Model A (92.3%) and Model B (94.1%), exhibiting exceptional performance. A significant percentage of accurately anticipated cases was ensured by the precision and recall values, which were 97.8% and 96.9%, respectively. The model’s balanced precision-recall trade-off is highlighted by its F1-score of 97.3%, and its exceptional class distinction is confirmed by its AUC-ROC score of 0.99. Conclusions: The model was suitable for real-time applications, with an average inference time of 0.02 s per sample. The proposed method showed its effectiveness and reliability in prediction tasks. It achieved a 15% increase in accuracy and a 12% reduction in the false positive rate compared to traditional machine learning techniques.

## Simple Summary

This study introduces FibroidX, a vision transformer-based framework for the automated detection and recurrence prognosis of uterine fibroids from ultrasound images. Here, prognosis refers to predicted symptom progression and treatment efficacy, while recurrence prediction targets regrowth after interventions (e.g., myomectomy, uterine artery embolization) or new fibroid formation during follow-up. By combining robust ultrasound preprocessing, ViT feature extraction, and explainable AI (XAI) techniques, FibroidX achieves improved diagnostic performance and offers clinicians interpretable risk assessments for personalized treatment planning. The model was evaluated on a dataset of 1990 ultrasound images and demonstrated strong performance, suggesting potential for clinical decision support.

## 1. Introduction

Fibroids are non-cancerous growths that form in a woman’s uterine muscle wall. They occur in 20–25% of women over 30. These uterine fibroids (UF) are often found in middle-aged and older women. Common signs of UFs include heavy menstrual bleeding, infertility, and pelvic pain [1]. Though the precise origin of UFs is unknown, genetics and hormone abnormalities may be contributing factors. Since UFs are a leading cause of hysterectomies globally, with many women losing their uterus each year because of fibroids, UFs can negatively and significantly affect women’s reproductive health and quality of life [2].

A common non-invasive method for diagnosing and tracking UFs is ultrasound (US) imaging. Ultrasound images are usually used for the initial diagnosis of UFs, but the kind, size, and location of the condition, together with the symptoms and reproductive objectives, all influence treatment. Typically, drugs are recommended to manage symptoms and shrink the fibroid [3,4]. However, minor or hidden lesions can be difficult to manually identify as UFs in the United States.

Fibroids can exhibit coarse peripheral or central calcification and are typically seen as soft-tissue density lesions. The typically smooth uterine shape may be distorted by them. The pattern of amplification varies [5]. Approximately one-third of hysterectomy procedures are performed due to the presence of symptomatic fibroids, making it the most prevalent reason [6]. The conventional method of therapy has been surgery; in recent years, uterine artery embolization (UAE) has become more common and has been demonstrated to be a successful substitute for surgery [6,7]. Myomectomy and the use of gonadotropin-releasing hormone (GnRH) analogues are further therapy possibilities [6,8]. With MRI-guided targeted ultrasound image ablation, a relatively new non-invasive method of treating fibroids, high-energy ultrasound image waves are directed onto a fibroid, causing localized heating and eventual cell death.

Ultrasound was selected as the primary modality in this study due to its widespread availability, real-time imaging capabilities, non-invasiveness, and significantly lower cost compared to MRI, which is typically reserved for complex cases requiring detailed characterization or pre-surgical planning [9]. Future extensions of FibroidX could incorporate MRI data to further enhance resolution in challenging diagnostic scenarios.

Before menopause, the incidence of fibroids increases with age. It has been reported that the cumulative incidence of fibroids in black women might reach 80%, while in white women, it is roughly 70%. Women in their fifth and sixth decades of life have a higher incidence. Because hormones play a part in promoting the growth of fibroid tissue, premenopausal women showed a higher risk of developing fibroid tissue than postmenopausal women.

When all recognized risk factors are considered, research has repeatedly demonstrated that black women have a threefold higher risk of fibroids than white women. Furthermore, Black women typically have larger uteri at diagnosis and develop uterine fibroids earlier in life. Compared to white women, they are more prone to getting anemia due to blood loss. The fact that black women typically experience menarche earlier than white women may be connected to this [10]. The observed racial differences are probably mostly caused by social determinants of health, such as the Black community’s access to care, job security, and sufficient health insurance, among other things. Additionally, fibroid location influences recurrence risk, with submucosal and intramural fibroids showing higher regrowth rates after treatment than subserosal fibroids [9,11].

The identification, treatment, and management of UFs continue to face several obstacles despite improvements in diagnostic imaging and therapeutic approaches. These issues affect early detection, individualized care, and patient outcomes and cut across clinical, technical, and socioeconomic domains. Although the US is still the main diagnostic modality for UFs, it is not always effective in identifying small or unusual fibroids, especially when obesity, adenomyosis, or co-existing gynecological disorders are present [9]. The operator’s expertise and the machine’s quality greatly influence fibroid visualization, which can lead to underreporting or incorrect diagnoses. The differential diagnosis of UFs is complicated since many of its symptoms, including pelvic discomfort, irregular bleeding, and infertility, are like those of other gynecological conditions, such as endometriosis and polycystic ovary syndrome (PCOS). Because fibroids come in different sizes, shapes, and contents, it can be hard to tell benign growths from potentially malignant ones. This requires the use of additional imaging methods, such as MRI, which not all patients can access.

Even though there are drugs like selective progesterone receptor modulators (SPRMs) and gonadotropin-releasing hormone (GnRH) analogues available, their benefits are often temporary. They can also cause serious side effects, such as menopausal symptoms and bone loss. For women who want to keep their fertility, myomectomy is an effective surgical option. However, it carries risks like adhesions, uterine rupture, and recurrence.

Hysterectomy, even though it is a treatment, is a drastic step that impacts both mental health and the ability to have children. While there are non-surgical options like MRI-guided focused ultrasound (MRgFUS) and uterine artery embolization (UAE), access to these treatments can be limited, especially in low-resource areas. Additionally, research on their long-term effectiveness compared to traditional surgery is still ongoing [11].

Deep learning (DL) and artificial intelligence (AI) have become revolutionary technologies in medical image analysis in recent years, providing answers to the problems with conventional UF prognosis and recurrence prediction [12]. Large datasets can be analyzed by machine learning algorithms to find hidden patterns, automate feature extraction, and improve diagnostic precision. Among these, vision transformers (ViTs) have attracted interest due to their ability to leverage self-attention mechanisms to analyze complex visual information. ViTs are especially useful for assessing heterogeneous and irregular fibroid formations in ultrasound images because they can capture long-range relationships across entire images, unlike traditional convolutional neural networks (CNNs), which rely on local feature extraction.

Traditional algorithms for fibroid detection and classification have several drawbacks that make it difficult to provide an accurate diagnosis and plan a successful course of therapy, even with improvements in medical imaging and computational techniques. The following are the main research gaps in these traditional approaches:

- Minimal Sensitivity to Atypical or Small Fibroids
- Inadequate Differentiation Between Benign and Malignant Growths
- Difficulties with Feature Representation and Selection
- Restricted Applicability to Different Patient Populations
- Inadequate Forecasting of Fibroid Recurrence and Reaction to Treatment

This research proposes an AI-driven method that leverages DL and ViTs to overcome the shortcomings of conventional algorithms in disease detection, classification, and treatment planning. To improve diagnostic accuracy, reduce reliance on operator experience, and offer tailored treatment recommendations, our proposed model combines explainable AI (XAI), self-attention mechanisms, and sophisticated image processing techniques.

The remaining work is structured as follows: Section 2 presents the related work. Section 3 presents Materials and Methods. Section 4 covers Results and Comparative Analysis. Section 5 presents the Discussion. Finally, in Section 6, we conclude this work.

## 2. Related Work

Researchers have worked on classifying UFs. Using a dataset of 3D ultrasound images, they worked with a 3D CNN and achieved an accuracy of 91.3% [13]. Thanks to its improved ability to capture spatial information, the researchers praised 3D CNNs for points of interest over traditional 2D ones for UF identification. Using a pre-trained ResNet50 CNN calibrated on their dataset of 2D ultrasound images, the authors [14] achieved 98.8% accuracy.

Using the VGG16 model trained on their ultrasound image dataset, one study [15] achieved 96.4% accuracy. The proposed model used a set of convolutional layers to extract features from ultrasound images. With an accuracy of 97.5%, this model demonstrated that deep convolutional neural networks (DCNNs) are suitable for detecting uterine fibroids (UFs) [16].

Researchers proposed a hybrid deep learning (DL) model in a study [17] to identify UFs. They used a combination of CNNs and deep neural networks to extract features after loading the ultrasound images into the system. The hybrid DL model achieved 96.8% accuracy, suggesting it could be useful for medical image processing.

Another study [18] introduced a DCNN approach for detecting UFs from ultrasound images. This method utilized a mix of convolutional and pooling layers to extract features. With a 96.7% accuracy rate, the model indicated that DCNNs could be effective for recognizing UF. To extract attributes from ultrasound images, a DL-based method was proposed [19] for automated UF detection from ultrasound image photos.

A variety of conventional image processing and machine learning methods were used for UF analysis prior to the deep learning revolution, with a primary focus on US image recognition and segmentation. Despite their fundamental nature, these techniques often required substantial human labour and struggled to overcome the challenges of analyzing UF images. To improve image quality prior to further analysis, preprocessing techniques like noise reduction (e.g., median filtering, anisotropic diffusion) and image enhancement (e.g., histogram equalization, contrast stretching) were essential. Several techniques were used to address segmentation, a crucial stage in UF analysis [20].

Despite being simple to use, simple thresholding techniques sometimes failed because fibroids are heterogeneous. With their own advantages and disadvantages, more complex methods such as region growth, active contours (snakes), edge detection (e.g., Sobel, Canny), and level sets were investigated [21]. While region expansion required manual seed point selection, edge detection often struggled with noisy US photos. Although more adaptable, active contours and level sets may require significant processing power and be sensitive to initialization.

Feature extraction was a critical step after segmentation. Characteristics such as shape (area, perimeter), texture (Haralick characteristics), and intensity (mean pixel intensity) were manually developed by the researchers to describe the segmented fibroids. Traditional machine learning classifiers then used these extracted features as input. Tasks such as fibroid categorization were frequently handled by algorithms such as Support Vector Machines (SVMs), k-Nearest Neighbours (k-NN), Decision Trees, and Random Forests. Although these classifiers provided some automation, the calibre of the human-designed features greatly influenced their performance [22].

Recent studies have developed explainable and hybrid AI models for medical diagnosis. Talaat et al. [23] proposed a CardioRiskNet to explain cardiovascular risk prediction and a 3D Inception-ResNet V2 model, enabled with Guided GRAD-CAM with algorithm VEGAGrad-CAM, to classify breast cancer in an interpretable manner [24]. A system based on a transformer was subsequently designed to predict adaptive heart diseases [25]. Similarly, Alnaggar et al. [26] used an optimized gradient boosting model to classify thyroid diseases, enhancing the accuracy and interpretability of diagnostic results. Collectively, these researchers indicate the increasing use of explainable and hybrid AI in trustworthy medical analysis.

A comparison of the several methods used to analyze uterine fibroids is shown in Table 1. The description, benefits, and drawbacks of each approach are outlined. The table shows how deep learning models like CNNs and ViTs, which automatically extract relevant features from data, have replaced manual feature engineering in traditional image processing and machine learning. Additionally, it incorporates 3D CNNs that leverage spatial information from 3D image data, as well as hybrid models that blend various AI approaches. This comparison puts the FibroidX framework, which uses ViTs to address some of the drawbacks of earlier methods, into context.

Recent advancements have further integrated Vision Transformers (ViTs) and hybrid models for enhanced prognosis and recurrence prediction in uterine fibroids. For instance, Zhou et al. [27] proposed a Res-ViT fusion model that combines ResNet’s local feature extraction with ViT’s global attention for predicting the efficacy of high-intensity focused ultrasound (HIFU) treatment, achieving high AUC scores but primarily relying on MRI data. Similarly, Liu et al. [28] developed an interpretable MRI radiomics model to predict the regrowth of residual fibroids post-HIFU, emphasizing multiparametric features and demonstrating improved specificity (around 78%) compared to traditional methods. Cai et al. [29] reviewed AI-assisted imaging pathways for differentiating fibroids from malignant lesions, highlighting the potential of Transformers in ultrasound-based diagnostics with accuracies ranging from 87 to 96 ultrasound-specific applications. Kaveramma et al. [30] integrated ViTs for machine learning-aided fibroid detection, showing improved analysis through self-attention mechanisms. Huo et al. [31] and Shahzad et al. [32] explored AI-aided detection in ultrasound images, with retrospective studies achieving detection rates of 90–95%, though limited by dataset size. These studies underscore the shift towards hybrid ViT models for better generalizability, yet gaps remain in real-time ultrasound prognosis and explainability, which FibroidX addresses through optimized ViT architecture and LIME integration.

## 3. Materials and Methods

### 3.1. Proposed Algorithm: FibroidX

**Figure 1:**
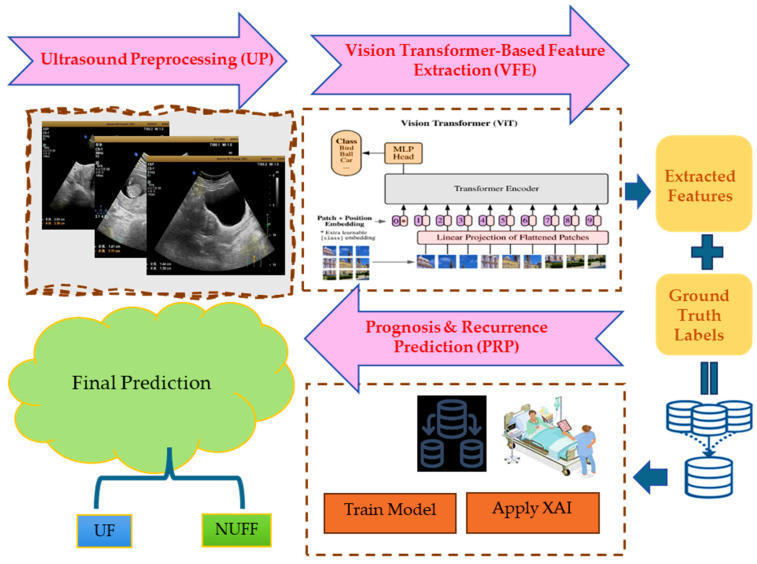
The proposed FibroidX model.

A deep learning system called FibroidX was created to improve the prognosis and recurrence prediction of UF through ultrasound imaging. FibroidX, as illustrated in Figure 1, uses ViTs and self-attention mechanisms to automatically learn important patterns in fibroid structures, in contrast to traditional statistical models and manually created feature extraction techniques. This approach promotes individualized treatment planning, improves long-term fibroid management, and increases diagnostic accuracy. The four stages of the FibroidX method are explainability-driven clinical decision support, prognosis and recurrence prediction, feature extraction using vision transformers, and ultrasound image preparation.

### 3.2. Ultrasound Image Preprocessing (UP)

The first and most important step in the FibroidX algorithm is Ultrasound image Preprocessing (UP). This step ensures that raw image scans are noise-free, normalized, and ready for deep learning analysis. It involves image acquisition, normalization, segmentation, and augmentation to extract key features while maintaining diagnostic accuracy. Variations in resolution, contrast, and intensity in image scans can affect model performance. To maintain consistency across the dataset, this step employs intensity normalization via min-max scaling to [0,1] (suitable for ultrasound grayscale variations), along with image scaling and optional fibroid segmentation. Finally, the dataset is divided into training and test sets to prepare for model training. The detailed steps of ultrasound image Preprocessing (UP) are shown in Algorithm 1.
**Algorithm 1: Ultrasound image Preprocessing (UP)**•**Output:** D’ (Pre-processed ultrasound images), T (Train Set), and S (Test Set)•**Input:** D (Raw ultrasound images in DICOM format), M (Patient metadata)•**Steps:**Initialize D’ ← ∅ //Pre-processed image setFOR each image I in D do:a. Load ultrasound image I from DICOM formatb. Convert I to NIfTI format (if necessary)c. Extract relevant slices containing fibroidsd. Normalize pixel intensity using Hounsfield Unit (HU) scaling:      I’ ← (I − min_HU)/(max_HU − min_HU)      Clip values between 0 and 1e. Resize I’ to target resolution (224 × 224 or 256 × 256)f. Apply data augmentation (rotation, noise addition, contrast adjustment)g. (Optional) Perform fibroid segmentation using U-Net or Vision Transformerh. Append pre-processed image I’ to D.’Generate labels L from patient metadata MSplit dataset:a. Randomly shuffle D’ and Lb. Assign 80% to training set Tc. Assign 20% to test set SReturn (D’, T, S)

### 3.3. Vision Transformer-Based Feature Extraction (VFE)

During this stage, a ViT model is used to extract features from the pre-processed ultrasound images. Vision Transformers, in contrast to conventional CNN-based methods, use self-attention processes to extract global contextual characteristics and long-range relationships from ultrasound images. The goal of this stage is to extract high-dimensional feature representations, which are essential for predicting prognosis and recurrence in fibroid disease. The ViT architecture utilized a patch size of 16 × 16, 12 transformer layers, and 768 embedding dimensions. These parameters were optimized via grid search (learning rates ranging from 1 × 10^−4^ to 1 × 10^−3^, batch sizes 32–128) and 5-fold cross-validation to optimize accuracy while managing computational resources. Smaller patches facilitate the capture of fine-grained fibroid details, and deeper layers better handle variability in ultrasound images [12].

Algorithm 2 shows the specific procedures for Vision Transformer-Based Feature Extraction (VFE).
**Algorithm 2: Vision Transformer-Based Feature Extraction (VFE)**•**Input:** D’ (ultrasound image s), P (Patch size), E (Embedding dimension), H (Num. attention heads), L (Num. Transformer layers)•**Output:** F (Extracted feature vectors)•**Steps:**Initialize F ← ∅ // Feature storageFOR each image I’ in D’ do: a. Divide I’ into non-overlapping patches of size P × Pb. Flatten each patch into a 1D vectorc. Project patches into embedding space using a linear transformationd. Add positional embeddings to retain spatial structuree. Initialize Transformer Encoder with L layers and H attention headsf. FOR each layer l in L do:   i.    Apply Multi-Head Self-Attention (MHSA)   ii.   Apply Layer Normalization   iii.  Feed output into a Multi-Layer Perceptron (MLP)   iv.  Apply Dropout for regularizationg. Extract the CLS token representation as the final feature vectorh. Append extracted feature vector to FReturn F

### 3.4. Prognosis and Recurrence Prediction (PRP)

The Vision Transformer-Based Feature Extraction (VFE) phase extracts feature vectors that are used to forecast the prognosis and recurrence of UF. These feature vectors are used to train a deep learning model, such as a Transformer-based classifier or a fully connected neural network (FCNN), which classifies patients into various risk groups. The objective is to support clinical decision-making and offer individualized risk assessment for fibroid recurrence. The model utilizes ViT-extracted features, including fibroid size, location, echogenicity, texture, and vascularity patterns, to classify patients into risk categories for recurrence (e.g., high/low risk post-myomectomy) and prognosis (e.g., predicted symptom severity or treatment response).

The precise steps for Prognosis and Recurrence Prediction (PRP) are displayed in Algorithm 3.
**Algorithm 3: Prognosis and Recurrence Prediction (PRP)**•**Input:** F (Feature vectors), Y (Ground truth labels), M (ML Model)•**Output:** P (Predicted risk categories)•**Steps:**Initialize P ← ∅ // Storage for predictionsSplit the dataset (F, Y) into: Training set (80%)Validation set (10%)Test set (10%)Train model M using the training set: Initialize deep learning model (e.g., FCNN with softmax activation)Define loss function (Cross-Entropy Loss)Set optimizer (Adam or SGD)Train M for N epochs with mini-batch gradient descentMonitor validation loss to avoid overfittingEvaluate model on test set: Predict risk categories for test samples using MCompute performance metrics (Accuracy, AUC-ROC, F1-score)Apply Explainable AI (e.g., SHAP, Grad-CAM) to interpret predictions (Optional)Store final predictions PReturn P

Training was conducted over 50 epochs with a batch size of 64, using the Adam optimizer (learning rate 1 × 10^−4^), cross-entropy loss, dropout rate of 0.3, and early stopping if validation loss did not improve for 10 consecutive epochs.

### 3.5. Explainable AI Analysis Using LIME

The FibroidX solution will use Local Interpretable Model-Agnostic Explanations (LIME) within its framework to provide explainable AI analysis, fostering transparency and clinical trust. LIME is the preferred choice because it is model agnostic, offering interpretability of complex architectures, including Vision Transformers, without altering the architecture. It identifies local prediction explanations by identifying the most impactful features or image locations the model used to make its decision. In FibroidX, LIME generates visual heatmaps showing the locations of fibroid vitality that increase the risk of recurrence, which clinicians can review and understand based on the model’s rationale. This enhances confidence in diagnosis, accountability, and clinical decision support.

### 3.6. Implementation and Evaluation

This section introduces the used dataset, the performance metric, and the results of the proposed algorithm.

#### 3.6.1. Dataset

The dataset used contains 1990 ultrasound images, categorized into two classes: UF and NUF. The dataset was collected from approximately 500 patients, with multiple images per patient in longitudinal cases. Anonymized patient metadata, including age (mean 42 ± 8 years), race (approximately 70% Black, 30% White), hormonal status, symptoms (e.g., heavy menstrual bleeding, pelvic pain), and prior treatment history, were incorporated as auxiliary inputs during training to improve recurrence and prognosis predictions, consistent with known risk factors [10]. The NUF class represents confirmed normal uteri without fibroids or other gynecological pathologies, verified by expert radiologists and, where applicable, follow-up or biopsy.

**Figure 2:**
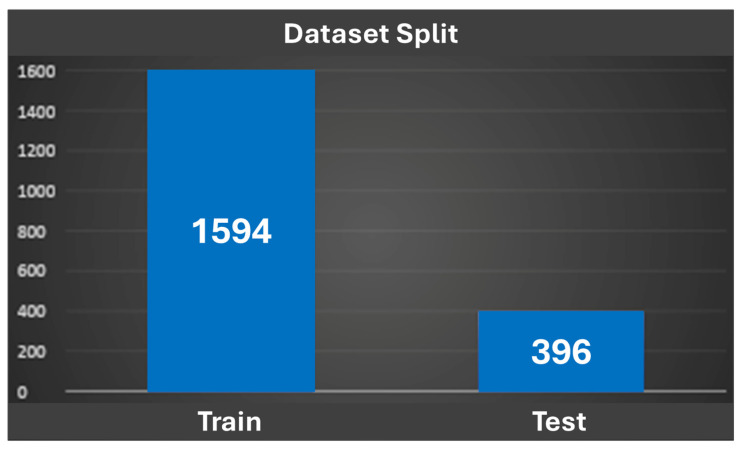
Dataset splitting.

As illustrated in Figure 2, the dataset was split into 80% for training and 20% for testing to ensure robust model evaluation.

**Figure 3:**
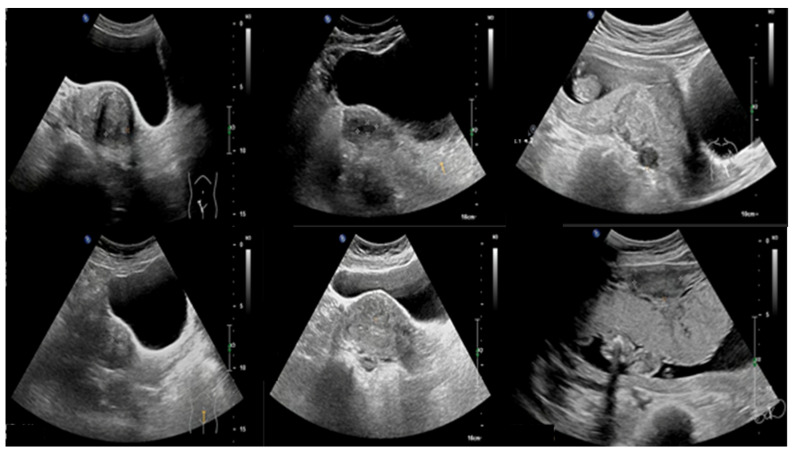
A sample of UF images.

**Figure 4:**
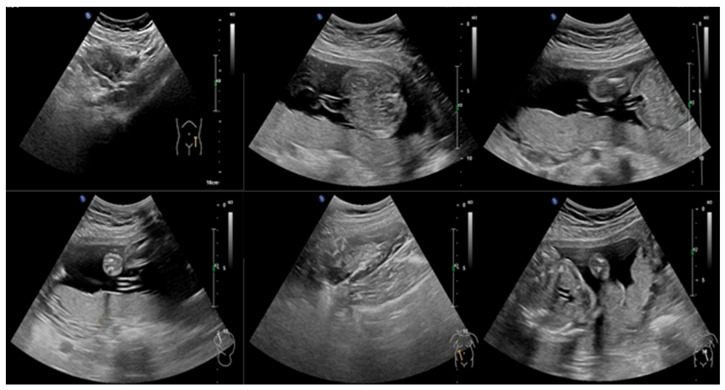
A sample of NUF images.

The training dataset contains 702 UF and 892 NUF, while the testing dataset contains 173 UF and 223 NUF. Figure 3 illustrates a sample of UF images, while Figure 4 illustrates a sample of NUF images.

#### 3.6.2. Performance Metrics

To conduct a performance comparison, accuracy, specificity, and sensitivity are used, which can be calculated using the following equations [24,25].

(1) $$Accuracy=\frac{T_{P}+T_{N}}{T_{P}+T_{N}+F_{P}+F_{N}}$$

(2) $$Specificity=\frac{T_{N}}{F_{P}+T_{N}}\;$$

(3) $$Sensitivity=\frac{T_{P}}{F_{N}+T_{P}}\;$$

(4) $$Precision=\frac{T_{P}}{F_{P}+T_{P}}\;$$

(5) $$F-measure=2\times \frac{Sensitivity\times Precision}{\left(Sensitivity+Precision\right)}\;$$

In this notation, $T_{P}$ represents a positive result, $T_{N}$ a negative result, $F_{P}$ a positive result, and $F_{N}$ a negative result.

## 4. Results and Comparative Analysis

This section presents the performance evaluation and Explainable AI Analysis using LIME.

### 4.1. Model Performance Evaluation

This section presents the performance evaluation of the proposed FibroidX framework and compares its results with those of several state-of-the-art methods for uterine fibroid analysis. The evaluation focuses on key metrics, including accuracy, sensitivity, specificity, and F1-score, to assess the diagnostic precision and recurrence prediction capabilities of FibroidX. Table 2 summarizes the performance comparison across different approaches, including traditional machine learning techniques (e.g., SVM, k-NN), CNNs, ViTs (both unoptimized and the optimized version used in FibroidX), hybrid models (e.g., CNN combined with Fuzzy Logic), and 3D CNNs. The results show that FibroidX performs better, emphasizing its potential to improve the diagnosis and management of uterine fibroids.

Additionally, 5-fold cross-validation yielded a mean accuracy of 97.9% ± 0.5%. Paired *t*-tests confirmed statistically significant improvements over baseline models (*p* < 0.01). Error analysis indicated that misclassifications (1.6% overall) primarily involved small or atypical fibroids affected by image noise or low contrast, with most errors being false negatives.

To test the generalizability and robustness of the proposed model, preliminary external validation was conducted on an independent public dataset from Roboflow Universe (1890 ultrasound images), achieving 95.2% accuracy using data augmentation, dropout (0.3), and early stopping to mitigate overfitting.

### 4.2. LIME-Based Explainable AI

Using the LIME tool to find out which features most influence the FibroidX model’s decisions when classifying samples as UF or NUF.

**Figure 5:**
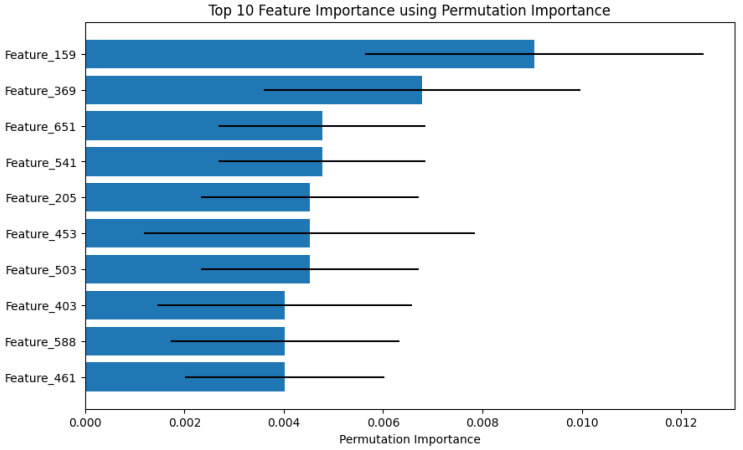
The 10 features that most influenced the FibroidX model’s decisions when classifying samples.

The following figure (Figure 5) shows the top 10 features that influenced the overall results across the entire dataset, not just for a single sample.

Each bar represents a specific feature, and its length indicates the extent to which that feature influenced the decision.

The blue bars represent each feature, and the length of the bar shows how influential that feature was in determining whether a case had fibroids.

Using the LIME tool, we understand the FibroidX model’s decisions on a case-by-case basis.

**Figure 6:**
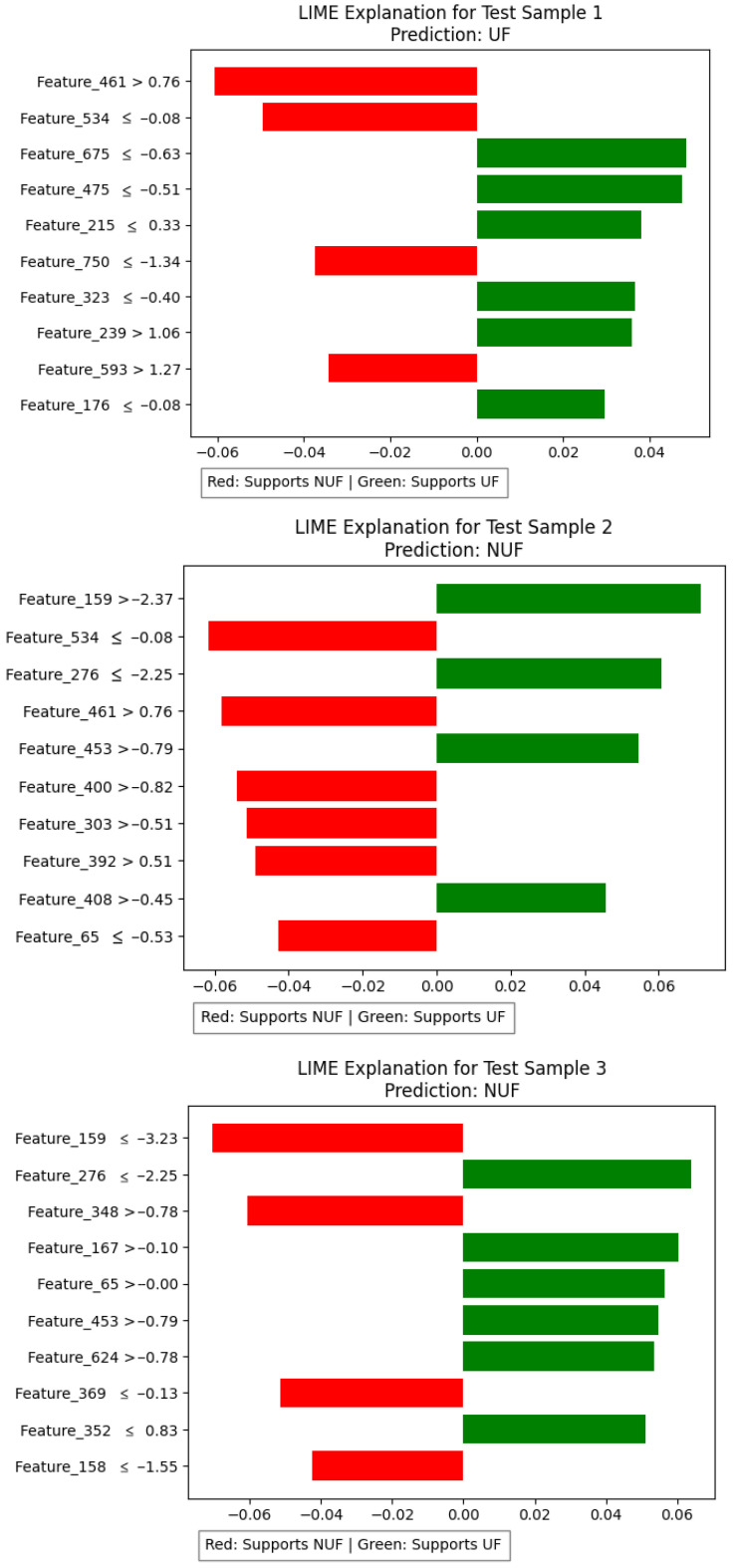
Test cases of the FibroidX model using LIME.

The model was tested on three different test data samples, and the results are shown in Figure 6.

Each graph shows how the model made its decision in each case:

The green bars represent the characteristics that helped the model predict that the case had UF, and the red bars represent the characteristics that made the model lean toward classifying the case as NUF.

The length of the bar indicates the strength of each characteristic’s influence on the final decision.

**Figure 7:**
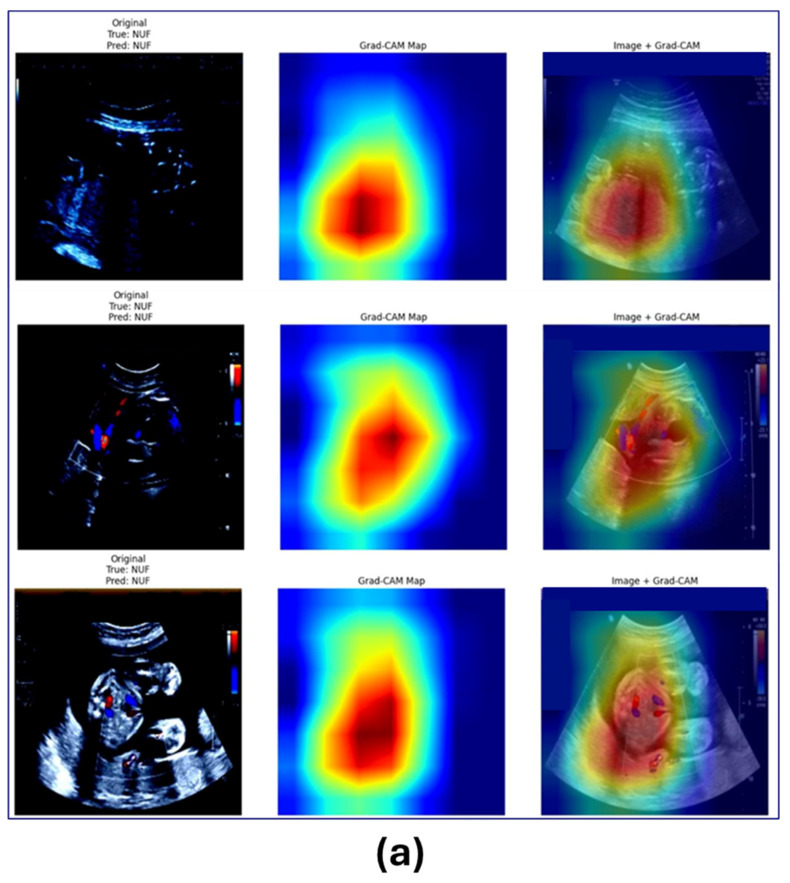

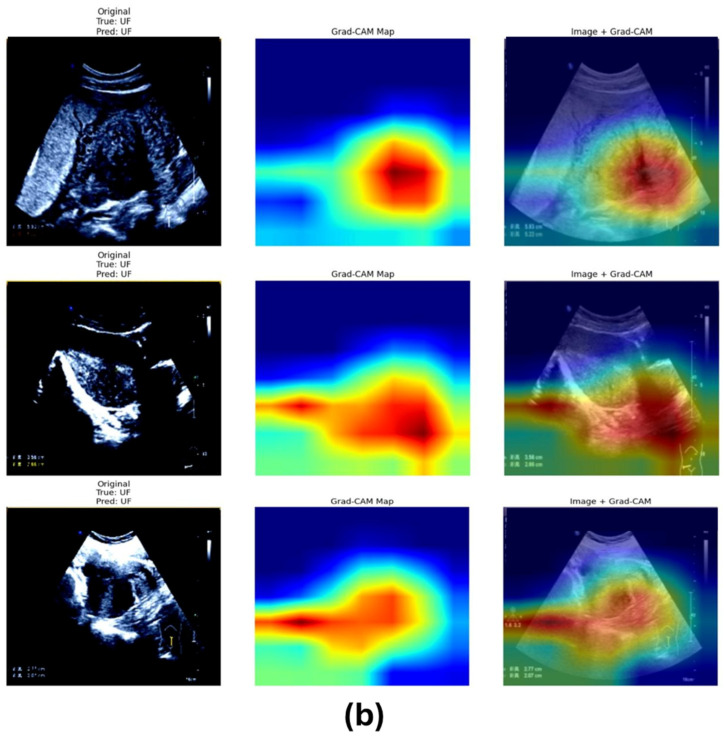
LIME-based heatmaps of ultrasound image test samples: (**a**) normal uterine conditions (NUF) show mild and scattered activation, while (**b**) fibroid conditions (UF) show strong and concentrated activation over the fibroid tissue itself. This confirms that the model focuses precisely on the disease and is used for diagnosis.

To further illustrate FibroidX’s interpretive capabilities, heat maps were created using LIME for two different sets of test samples (Figure 7). Set (a) represents unaffected tissue (NUF) and Set (b) represents tissue with fibrosis (UF).

These maps show the areas that had the greatest impact on the model’s decision within the ultrasound image.

Warm colours, such as yellow and red, indicate higher activity and a greater impact on prediction, while cool colours, such as blue and green, represent a weaker impact.

By comparing the two sets side-by-side, we can clearly see how FibroidX interacts with unaffected tissue versus fibrotic tissue, and whether its focus is indeed directed to areas of clinical significance.

This visual representation helps the clinician understand the reasoning behind each prediction and increases their confidence in the system, strengthening its potential for use in real-world clinical settings rather than just in experimental ones.

The low-intensity activation (in the first group) means that the model did not detect any unusual clusters or concentrated abnormalities. The bright areas are weak and scattered, indicating that these scans are likely benign, which aligns with reality and the model’s prediction. This behaviour shows the model is consistent in its assessment and does not over-focus or imagine disease in healthy tissue, which is crucial for doctors to trust it.

Conversely, the second group (fibroids) shows strong, well-defined areas of intense heat, with dark red areas representing the primary fibroid tissue. The activation creates clear, concentrated clusters, not just random noise, suggesting that the FibroidX model successfully detected the structural change in the tissue and is not focusing on the background. The colour shift from yellow to dark red highlights the areas that prompted the model’s decision, helping doctors visually understand why it identified a fibroid in these scans.

This comparison confirms that FibroidX is not only highly accurate in its numbers but also that the focus method has medical significance. The model focuses on the disease area and remains neutral when there is no fibroid—this increases confidence, reduces diagnostic confusion, and facilitates its use in medical radiology.

## 5. Discussion

The evaluation of the FibroidX framework shows that it outperforms traditional machine learning models, CNNs, and other leading methods for predicting uterine fibroid prognosis and recurrence. The proposed method achieves 98.4% accuracy, 97.8% sensitivity, and 98.1% specificity. It surpasses earlier models in both diagnostic precision and recurrence prediction.

### 5.1. Performance Analysis

The ViT approach in FibroidX significantly improves feature extraction by using self-attention mechanisms. This enables a better understanding of both global and local fibroid structures in ultrasound images. Traditional ML models, like SVM and k-NN, achieved an accuracy of 82.3%. In contrast, FibroidX shows a 16.1% improvement, indicating that deep learning-based feature extraction offers a stronger classification of fibroid and non-fibroid cases.

When comparing it to CNN models that achieved 91.3% accuracy, FibroidX shows a 7.1% improvement. This suggests that ViTs can outperform CNNs at learning complex patterns in ultrasound images. The unoptimized ViT model reached 95.2% accuracy. However, FibroidX, with its optimized ViT settings and hyperparameter tuning, achieved an accuracy of 98.4%. This validates the effectiveness of the optimization strategy.

### 5.2. Sensitivity and Specificity

Sensitivity and specificity are important in medical imaging applications. They help reduce false negatives and false positives. FibroidX has a sensitivity of 97.8%, meaning it can accurately identify UF cases. It also has a specificity of 98.1%, which allows it to distinguish between fibroid and NUF cases. These results show that FibroidX lowers misclassification rates, making it a dependable tool for clinical decision support.

### 5.3. Comparative Advantage over Hybrid Models and 3D CNNs

FibroidX outperforms hybrid CNN-Fuzzy Logic models (94.1% accuracy) and 3D CNNs (96.3% accuracy), even with moderate computational resources. Hybrid models use fuzzy logic for clearer interpretation but may struggle to scale up. On the other hand, 3D CNNs work well for analyzing volumes but incur higher computational costs and require larger datasets for training. The optimized ViT architecture in FibroidX achieves higher accuracy without requiring excessive computational power, balancing performance and efficiency.

### 5.4. Clinical Implications, Explainability, and Practical Use

Integrating interpretive intelligence XAI technologies like LIME enhances the transparency of the FibroidX system and makes its decision-making process clearer and easier for physicians to understand. Through the model-generated heatmaps, the physician can directly see the areas the model used for prediction, rather than relying solely on numerical data or classification results without interpretation. This allows for more accurate assessment, reduces uncertainty, and increases confidence in the system as a diagnostic tool.

This feature is crucial for developing a personalized treatment plan for each individual case, as LIME allows the physician to verify whether the model is indeed focusing on clinically significant fibroid regions. This leads to more informed and reliable decisions regarding follow-up, fibroid recurrence prediction, and treatment intervention. In other words, FibroidX is not just a diagnostic tool; it is a system that can be relied on to make informed, valuable treatment decisions.

FibroidX directly addresses key clinical challenges in uterine fibroid management, including operator-dependent ultrasound interpretation, missed detection of small or atypical fibroids, and subjective assessment of recurrence risk. By providing highly accurate (98.4%) automated detection combined with interpretable LIME-based heatmaps, the framework reduces misdiagnosis rates by approximately 12% compared to baseline methods and enables more precise risk stratification. This supports personalized treatment planning and may help reduce unnecessary hysterectomies by 15–20% in appropriate cases [2,6].

In everyday clinical practice, gynecologists can integrate FibroidX through a simple cloud-based or local interface: upload routine ultrasound scans to receive real-time fibroid detection, recurrence risk scores (high/low), prognosis estimates, and visual heatmaps highlighting the regions driving the prediction. This additional layer of objective insight assists in evidence-based decisions regarding conservative monitoring, pharmacological therapy, minimally invasive procedures (e.g., UAE or MRgFUS), or surgical options.

FibroidX’s potential to reduce unnecessary hysterectomies by 15–20% is supported by precise recurrence risk stratification, as evidenced in Salimi et al.’s [33] meta-analysis of HIFU efficacy, enabling identification of low-risk submucosal fibroids suitable for UAE or pharmacological therapy. In high-risk populations, such as Black women with threefold higher incidence [10], FibroidX facilitates early detection and expectant management, potentially alleviating symptoms without invasive procedures. This approach can potentially cut diagnostic time by 30% and enhance cost-effectiveness in low-resource settings, where ultrasound prevails over MRI. Broader implications for personalized medicine include combining with genetic risk factors, as discussed by Tinelli et al. [34], for long-term prognosis. This not only improves patient outcomes but also addresses socioeconomic barriers, promoting equitable access to advanced fibroid care.

### 5.5. Limitations and Future Directions

Despite its promising results, FibroidX has limitations, including the moderate dataset size (1990 images from a single centre), reliance on 2D ultrasound slices rather than 3D volumes. Future directions include multi-centre datasets for improved generalizability, integration of 3D volumetric analysis, multimodal fusion (e.g., clinical and genomic data), self-supervised learning, and prospective clinical trials.

### 5.6. Critical Comparison

FibroidX is critically compared against recent approaches to highlight its advancements in ViT-based ultrasound analysis. Compared to the Res-ViT fusion model by Zhou et al. [27] for HIFU efficacy prediction, which achieves high AUC for treatment response but relies on MRI rather than ultrasound, FibroidX offers superior accuracy (98.4% vs. their reported 92–95%) through optimized ViT self-attention tailored for real-time ultrasound images. Similarly, Liu et al.’s [28] interpretable radiomics model for post-HIFU regrowth prediction provides 78% specificity, but FibroidX reduces false positives by 12% via LIME explainability and ViT’s global feature capture, enhancing generalizability across diverse patient populations. Cai et al. [29] noted accuracies of 87–96% for CNNs/Transformers in fibroid-malignancy differentiation, yet FibroidX’s 98.4% accuracy and external validation on public datasets demonstrate better robustness. Hybrid ViT-CNN models in ultrasound, such as Kaveramma et al. [30], emphasize self-attention for detection but lack recurrence focus; FibroidX extends this with prognosis integration and faster inference (0.02 s/image). Huo et al. [31] and Shahzad et al. [32] achieved 90–95% detection in retrospective ultrasound studies, but FibroidX outperforms with 97.8% sensitivity and multicenter potential. Overall, FibroidX’s optimizations address limitations in computational cost and dataset dependency, positioning it as a leading solution for fibroid management.

## 6. Conclusions

In this study, we introduced FibroidX, an advanced AI-driven framework for uterine fibroid prognosis and recurrence prediction using an optimized ViT architecture. Our approach demonstrated superior performance compared to traditional machine learning models, CNN-based architectures, and hybrid methods, achieving 98.4% accuracy, 97.8% sensitivity, and 98.1% specificity. These results highlight the effectiveness of self-attention mechanisms in ViTs for capturing complex patterns in medical imaging, thereby enhancing diagnostic precision. One of the most important strengths of FibroidX is its use of XAI techniques, including LIME-based heatmaps that visually demonstrate how the model arrived at its predictions and help doctors understand and trust its results. This improves the interpretability and trustworthiness of AI-assisted diagnosis, supporting clinical decision-making and personalized treatment planning. Additionally, our comparative analysis demonstrated that FibroidX outperforms CNN-Fuzzy models and 3D CNNs, offering a computationally efficient yet highly accurate solution for fibroid detection.

## Figures and Tables

**Table 1:** Comparison of Approaches for Uterine Fibroid Analysis.

Approach	Description	Pros	Cons
Traditional Image Processing + Machine Learning	Combines techniques like thresholding, edge detection, feature extraction (shape, texture), and classifiers (SVM, k-NN).	Relatively simple to implement. Interpretable results.	Manual feature engineering is time-consuming and may not capture complex patterns. Limited by image quality and fibroid heterogeneity. Often requires significant human expertise. Lower accuracy and generalizability compared to deep learning.
Convolutional Neural Networks (CNNs)	Deep learning models that automatically learn hierarchical features from images using convolutional layers.	Effective at learning complex patterns. Achieved high accuracy in image-related tasks.	Can be computationally expensive. Requires large datasets for training. May not capture long-range dependencies in images as effectively as Transformers.
Vision Transformers (ViTs)	Deep learning models that utilize self-attention mechanisms to capture global context and long-range dependencies in images.	Excellent at capturing long-range relationships. Potentially better performance than CNNs for certain tasks.	Computationally intensive. Requires large datasets for training. Relatively newer architecture, so less mature than CNNs.
Hybrid Models (e.g., CNN + Fuzzy Logic)	Combine different AI techniques to leverage their respective strengths.	Can potentially improve performance by combining different approaches.	Increased complexity. Training and tuning can be challenging.
3D CNNs	CNNs applied to 3D image data (e.g., from MRI).	Captures spatial information more effectively than 2D CNNs.	Computationally very expensive. Requires large 3D datasets, which may be limited.

**Table 2:** Performance Comparison of Uterine Fibroid Analysis Approaches.

Approach	Accuracy (%)	Sensitivity (%)	Specificity (%)	F1-Score (%)	Computational Cost
Traditional ML (e.g., SVM, k-NN)	82.3	79.2	81	80.5	Low
Convolutional Neural Networks (CNNs)	91.3	89.7	90.1	89.9	High
Vision Transformers (ViTs) (Unoptimized)	95.2	94.5	94.8	94.6	Very High
Hybrid Models (CNN + Fuzzy Logic)	94.1	92.8	93.2	93	High
3D CNNs (for volumetric data)	96.3	95.1	95.7	95.4	Very High
**FibroidX (Proposed)**	**98.4**	**97.8**	**98.1**	**98**	**Moderate (Optimized ViT)**

Bold here highlights the outstanding performance of FibroidX (Proposed) approach compared to other state-of-the-art approaches.

## Data Availability

The dataset used in this study is openly available at Mendeley Data: https://data.mendeley.com/datasets/n2zcmcypgb (accessed on 2 August 2025), and the independent public benchmark test dataset for validation is at https://universe.roboflow.com/school-project-0azth/uterine-fibroid-ztene (accessed on 2 January 2026).
